# XPO1 expression worsens the prognosis of unfavorable DLBCL that can be effectively targeted by selinexor in the absence of mutant p53

**DOI:** 10.1186/s13045-020-00982-3

**Published:** 2020-11-04

**Authors:** Manman Deng, Mingzhi Zhang, Zijun Y. Xu-Monette, Lan V. Pham, Alexandar Tzankov, Carlo Visco, Xiaosheng Fang, Govind Bhagat, Feng Zhu, Karen Dybkaer, April Chiu, Wayne Tam, Youli Zu, Eric D. Hsi, William W. L. Choi, Jooryung Huh, Maurilio Ponzoni, Andrés J. M. Ferreri, Michael B. Møller, Benjamin M. Parsons, J. Han van Krieken, Miguel A. Piris, Jane N. Winter, Fredrick Hagemeister, Lapo Alinari, Yong Li, Michael Andreeff, Bing Xu, Ken H. Young

**Affiliations:** 1 Department of Hematology, The First Affiliated Hospital of Xiamen University and Institute of Hematology, Xiamen University, School of Medicine, Xiamen, Fujian China; 2 Division of Hematopathology, Department of Pathology, Duke University Medical Center, Durham, NC 27710 USA; 3 Department of Oncology, The First Affiliated Hospital of Zhengzhou University, Zhengzhou, China; 4 Phamacyclics, an Abbvie Company, San Francisco, CA USA; 5 Institute of Pathology, University Hospital Basel, Basel, Switzerland; 6 Department of Medicine, Section of Hematology, University of Verona, Verona, Italy; 7 Columbia University Medical Center and New York Presbyterian Hospital, New York, NY USA; 8 Aalborg University Hospital, Aalborg, Denmark; 9 Mayo Clinic, Rochester, MN USA; 10 Weill Medical College of Cornell University, New York, NY USA; 11 The Methodist Hospital, Houston, TX USA; 12 Cleveland Clinic, Cleveland, OH USA; 13 University of Hong Kong Li Ka Shing Faculty of Medicine, Hong Kong, China; 14 Asan Medical Center, Ulsan University College of Medicine, Seoul, Korea; 15 San Raffaele H. Scientific Institute, Milan, Italy; 16 Odense University Hospital, Odense, Denmark; 17 Gundersen Lutheran Health System, La Crosse, WI USA; 18 Radboud University Nijmegen Medical Centre, Nijmegen, The Netherlands; 19 Hospital Universitario Marqués de Valdecilla, Santander, Spain; 20 Feinberg School of Medicine, Northwestern University, Chicago, IL USA; 21 Department of Lymphoma/Myeloma, The University of Texas MD Anderson Cancer Center, Houston, TX USA; 22 Division of Hematology, Department of Internal Medicine, The Ohio State University, Columbus, OH USA; 23 Department of Medicine, Baylor College of Medicine, Houston, TX USA; 24 Department of Leukemia, The University of Texas MD Anderson Cancer Center, Houston, TX USA; 25 Key Laboratory of Xiamen for Diagnosis and Treatment of Hematological Malignancy, Xiamen, China; 26 Duke Cancer Institute, Durham, NC USA

**Keywords:** XPO1, DLBCL, HGBCL, *TP53* mutation, Selinexor, MYC, BCL2

## Abstract

The XPO1 inhibitor selinexor was recently approved in relapsed/refractory DLBCL patients but only demonstrated modest anti-DLBCL efficacy, prompting us to investigate the prognostic effect of XPO1 in DLBCL patients and the rational combination therapies in high-risk DLBCL. High XPO1 expression (XPO1^high^) showed significant adverse prognostic impact in 544 studied DLBCL patients, especially in those with BCL2 overexpression. Therapeutic study in 30 DLBCL cell lines with various molecular and genetic background found robust cytotoxicity of selinexor, especially in cells with *BCL2-*rearranged (*BCL2*-R^+^) DLBCL or high-grade B-cell lymphoma with *MYC*/*BCL2* double-hit (HGBCL-DH). However, expression of mutant (Mut) p53 significantly reduced the cytotoxicity of selinexor in overall cell lines and the *BCL2*-R and HGBCL-DH subsets, consistent with the favorable impact of XPO1^high^ observed in Mut-p53-expressing patients. The therapeutic effect of selinexor in HGBCL-DH cells was significantly enhanced when combined with a BET inhibitor INCB057643, overcoming the drug resistance in Mut-p53-expressing cells. Collectively, these data suggest that XPO1 worsens the survival of DLBCL patients with unfavorable prognostic factors such as BCL2 overexpression and double-hit, in line with the higher efficacy of selinexor demonstrated in *BCL2*-R^+^ DLBCL and HGBCL-DH cell lines. Expression of Mut-p53 confers resistance to selinexor treatment, which can be overcome by combined INCB057643 treatment in HGBCL-DH cells. This study provides insight into the XPO1 significance and selinexor efficacy in DLBCL, important for developing combination therapy for relapsed/refractory DLBCL and HGBCL-DH.

## To the editor

XPO1 (exportin 1) is a well-characterized nuclear export protein responsible for the nuclear-cytoplasmic transport and cellular homeostasis of up to 220 cargoes, including the tumor suppressors p53 and IκB [1, 2]. Abnormal XPO1 expression correlates with worse prognoses in human malignancies. Targeting XPO1 is a promising therapeutic approach in cancer [1, 2]. The XPO1 inhibitor selinexor has received FDA approval recently to treat refractory/relapsed (R/R) diffuse large B-cell lymphoma (DLBCL) after at least 2 lines of systemic therapy, showing an overall response rate of 28% in the SADAL trial [3]. However, it remains largely unknown whether and how XPO1 interplays with other adverse predictors in DLBCL, how to predict selinexor effectiveness, and what combination therapy is optimal in R/R DLBCL patients. Here, we evaluated the prognostic significance of XPO1 expression in 544 well-characterized DLBCL cases, and investigated the therapeutic effect of selinexor in 30 DLBCL cell lines with variable genetic background.

**Fig. 1:**
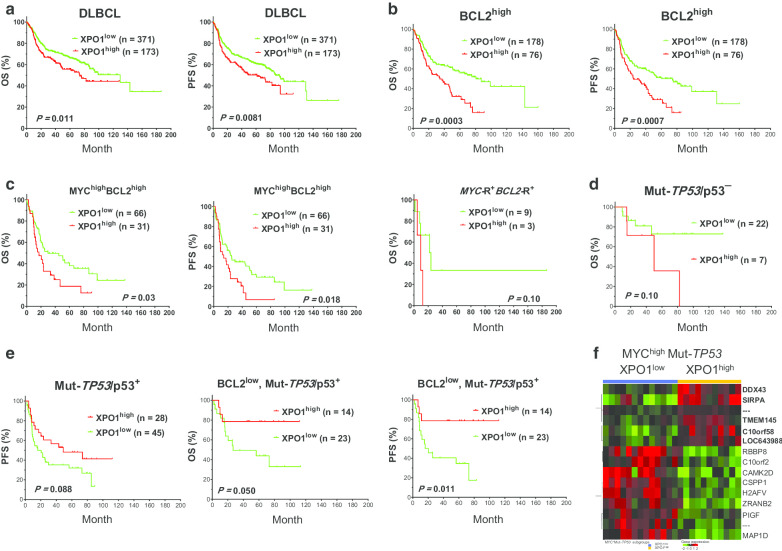
Impact of XPO1 expression on patient survival in DLBCL. **a** In the entire cohort, DLBCL patients with high level of XPO1 expression (XPO1^high^) had significantly worse OS and PFS than those with low or negative XPO1 expression (XPO1^low^). **b** XPO1^high^ remarkably worsened the OS/PFS of DLBCL patients with BCL2^high^ expression. **c** XPO1^high^ significantly worsened the OS/PFS of patients with dual MYC^high^BCL2^high^ expression, and showed a trend of unfavorable effect on OS in patients with dual *MYC*/*BCL2* rearrangements (*MYC*-R^+^*BCL2*-R^+^, HGBCL-DH). **d** In *TP53*-mutated (Mut) DLBCL patients without Mut-p53 overexpression, XPO1^high^ showed a trend of unfavorable prognostic effect on OS. **e** In Mut-*TP53* DLBCL patients with Mut-p53 overexpression, XPO1^high^ showed favorable prognostic effect, which was not significant in overall patients but significant in the subset with low BCL2 expression. **f** Significantly differentially expressed genes between XPO1^high^ and XPO1^low^ patients with concurrent Mut-*TP53* and MYC^high^

Patients and Methods for this study are detailed in Additional file 1. XPO1 expression was observed in 217 of 544 (40%) DLBCL patients with a mean level of 24%. High level of XPO1 expression (XPO1^high^; > 30%) predicted significantly poor progressive-free survival (PFS) and overall survival (OS) in DLBCL patients (Fig. 1a). DLBCL is classified into prognostic favorable germinal center B-cell-like (GCB) and unfavorable activated B-cell-like (ABC) subtypes [4]. XPO1^high^ significantly shortened the PFS/OS in ABC-DLBCL but not GCB-DLBCL (Additional file 1: Figure S1A–B). XPO1^high^ showed significant association with p53 overexpression (p53^+^) and dual p53^+^MYC^high^ expression but not clinical features (Additional file 1: Table S1), unlike a previous study using a different scoring system for XPO1 expression in 131 DLBCL patients [5].

Whether XPO1^high^ interacts with other adverse prognostic factors and whether XPO1 is a potential therapeutic target in high-risk DLBCL patients were further examined. XPO1^high^ remarkably worsened the OS and PFS of DLBCL with BCL2^high^ or dual MYC^high^BCL2^high^ expression (Fig. 1b,c), which is known as double-expressor lymphoma with unfavorable prognosis [6]. Trends of adverse impact were also observed on PFS in *MYC*-rearranged (R^+^) patients (*P* = 0.097; Additional file 1: Figure S1C) and OS in patients with dual *MYC-*R^+^*BCL2*-R^+^ (Fig. 1c) with dismal prognosis, defined as high-grade B-cell lymphoma with *MYC*/*BCL2* double-hit (HGBCL-DH) [7]. In patients with *TP53* mutation (Mut-*TP53*) [8], XPO1^high^ showed opposite prognostic effects in patients with and without Mut-p53 protein overexpression [9], suggesting the nuclear export may attenuate the oncogenic gain-of-function of Mut-p53. In contrast to the negative impact of XPO1^high^ in Mut-*TP53*/p53-negative patients (Fig. 1d) and in *TP53*-wild type (Wt-*TP53*) patients (Additional file 1: Figure S1D), a favorable effect was associated with XPO1^high^ in Mut-*TP53*/p53-positive patients, which was significant in the BCL2^low^ subset (Fig. 1e). Gene expression profiling [4] analysis identified a distinct gene expression signature for XPO1^high^ in patients with Mut-*TP53* and MYC^high^ (Fig. 1f), including upregulation of *SIRPA*, which encodes SIRPα, a receptor for CD47 transmitting “do not eat me” signal in phagocytosis, and downregulation of several genes related to DNA repair, metabolism, splicing, or biosynthesis (Additional file 1: Table S2).

**Fig. 2:**
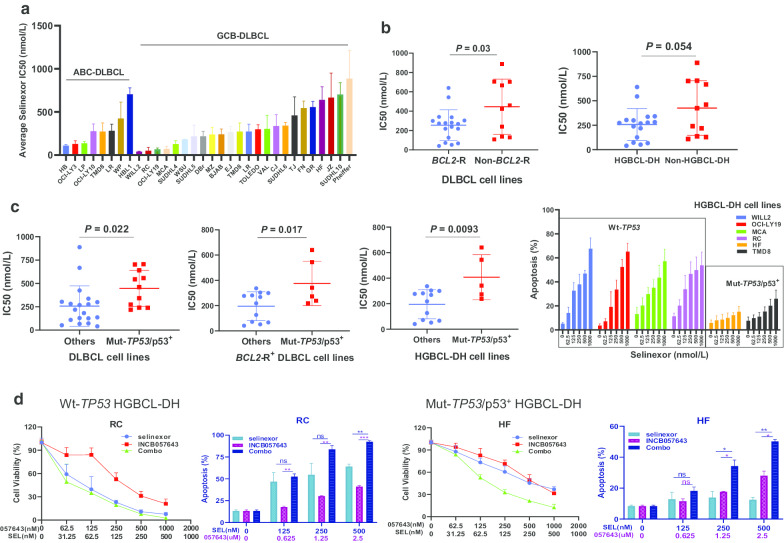
Therapeutic effect of selinexor alone or in combination with a BET inhibitor INCB057643 in DLBCL cellular models. **a** The effect of 72-h selinexor exposure on cell viability of 30 DLBCL cell lines. Waterfall graph showed the specific IC50 value of selinexor for each cell line with either ABC or GCB subtype of DLBCL. **b** DLBCL cell lines with *BCL2* rearrangement (*BCL2*-R) or HGBCL-DH were more sensitive to selinexor with a lower mean IC50 value compared with other cell lines. **c** The presence of mutant (Mut) p53 in DLBCL cells significantly reduced the cytotoxicity of selinexor, especially significant in HGBCL-DH cell lines. Selinexor promoted more significant apoptosis in Wt-*TP53* HGBCL-DH cells than in Mut-*TP53*/p53-expressing HGBCL-DH cells. **d** INCB057643 and selinexor were cooperative in reducing cell viability and inducing apoptosis in HGBCL-DH cells with Wt-*TP53* or Mut-*TP53*/p53^+^

Next, selinexor was assessed in 30 DLBCL cell lines, which resulted in significantly reduced cell viability with varying IC50 values (Fig. 2a). ABC-DLBCL and GCB-DLBCL cells were similarly vulnerable to selinexor (Additional file 1: Figure S1E), consistent with results in the SADAL clinical trial [3]. Biomarkers significantly associated with higher sensitivity (lower IC50) to selinexor cytotoxicity included *BCL2*-R and HGBCL-DH (Fig. 2b) but not *MYC*-R. In contrast, presence of Mut-*TP53*/p53^+^ significantly reduced the anti-lymphoma efficacy of selinexor, especially in HGBCL-DH cells (Fig. 2c; Additional file 1: Figure S1F).

Limited efficacy of selinexor in HGBCL with Mut-*TP53*/p53^+^ calls for combination strategy. Previous studies showed the synergy between selinexor and venetoclax in DLBCL and double-hit lymphoma [10, 11]. However, in the SADAL trial [3], patients with MYC^high^ (but not BCL2^high^) expression had a lower overall response rate than those without. MYC expression can be inhibited by targeting the bromodomain and extra-terminal domain (BET) proteins [12]. We therefore combined selinexor with a novel BET inhibitor INCB057643. Synergistic effect was observed in DLBCL/HGBCL cells, especially in HGBCL-DH cells with Mut-*TP53*/p53^+^ (Fig. 2d), supporting INCB057643/selinexor combination as a therapeutic option for HGBCL-DH patients.

In summary, this study demonstrates that XPO1^high^ is a valuable biomarker in DLBCL with unfavorable prognostic factors, predictive of significantly poorer outcomes in ABC-DLBCL, BCL2^high^ DLBCL, and double-expressor lymphoma but not Mut-p53-expressing DLBCL. Targeting XPO1 with selinexor is similarly effective in GCB-DLBCL and ABC-DLBCL cells, and remarkably effective in *BCL2*-R^+^ DLBCL and HGBCL cells without Mut-*TP53*/p53-positivity. In DLBCL/HGBCL cells, Mut-*TP53*/p53-positive expression predicts resistance to selinexor. INCB057643 synergizes with selinexor in HGBCL-DH cells, overcoming resistance in Mut-*TP53*/p53-positive HGBCL-DH. These findings warrant future investigation on the role of XPO1, selinexor, and combined BET inhibition in R/R DLBCL and HGBCL-DH.

## Supplementary information

**Additional file 1**.** Table S1**: Clinicopathologic and molecular characteristics of DLBCL patients with high or low XPO1 expression.** Table S2**: Significantly differentially expressed genes between XPO1^high^ and XPO1^low^ DLBCL patients with concurrent *TP53* mutation and high MYC expression.** Figure S1**: Biomarker study for XPO1 and selinexor. (A–B) XPO1^high^ expression showed significant adverse prognostic impact in the ABC subtype but not the GCB subtype of DLBCL. (C) XPO1^high^ expression showed a trend of unfavorable prognostic effect on PFS in *MYC*-rearranged (*MYC*-R^+^) DLBCL. (D) XPO1^high^ expression was associated with significantly poorer survival in DLBCL patients with wild type (Wt) *TP53*. (E) ABC-DLBCL and GCB-DLBCL cells showed similar sensitivity to the cytotoxicity of selinexor. (F) *TP53* mutation (Mut-*TP53*) significantly reduced the anti-lymphoma efficacy of selinexor in HGBCL-DH cells. IC50 values were calculated by GraphPad Prism 8 based on the cell viability data after 72-hour treatment.

## Data Availability

The datasets supporting the conclusions of this study are included in the figures and additional files.

## Abbreviations

DLBCL: Diffuse large B-cell lymphoma
R/R: Relapsed or refractory
GCB: Germinal center B-cell-like
ABC: Activated B-cell-like
PFS: Progressive-free survival
OS: Overall survival
*MYC*-R: *MYC* rearrangement
*BCL2*-R: *BCL2* rearrangement
HGBCL-DH: High-grade B-cell lymphoma with *MYC* and *BCL2* double-hit
Wt: Wild type
Mut: Mutant or mutated
BET: Bromodomain and extra-terminal domain
