# Supplementary material for: XPO1 expression worsens the prognosis of unfavorable DLBCL that can be effectively targeted by selinexor in the absence of mutant p53
Source: J Hematol Oncol. 2020 Nov 4;13:148. doi: 10.1186/s13045-020-00982-3 (PMC7641823; doi:10.1186/s13045-020-00982-3)
Supplement: Supplementary file 1 — Additional file 1. Table S1: Clinicopathologic and molecular characteristics of DLBCL patients with high or low XPO1 expression. Table S2: Significantly differentially expressed genes between XPO1high and XPO1low DLBCL patients with concurrent TP53 mutation and high MYC expression. Figure S1: Biomarker study for XPO1 and selinexor. (A–B) XPO1high expression showed significant adverse prognostic impact in the ABC subtype but not the GCB subtype of DLBCL. (C) XPO1high expression showed a trend of unfavorable prognostic effect on PFS in MYC-rearranged (MYC-R+) DLBCL. (D) XPO1high expression was associated with significantly poorer survival in DLBCL patients with wild type (Wt) TP53. (E) ABC-DLBCL and GCB-DLBCL cells showed similar sensitivity to the cytotoxicity of selinexor. (F) TP53 mutation (Mut-TP53) significantly reduced the anti-lymphoma efficacy of selinexor in HGBCL-DH cells. IC50 values were calculated by GraphPad Prism 8 based on the cell viability data after 72-hour treatment. [file 13045_2020_982_MOESM1_ESM.pdf]

SUPPLEMENTARY MATERIALS AND METHODS

Patients and samples

Total 544 patients with de novo DLBCL (including 12 HGBCL-DH cases with DLBCL morphology) uniformly treated with R-CHOP were included in this study. Diagnosis and classification were performed by hematopathologists based on 2008/2016 WHO classification [1,2]. Fluorescence in situ hybridization (FISH) was used to detect *MYC*/*BCL2* rearrangements and gene expression profiling (GEP, deposit in GSE#31312) were analyzed as described previously [3-6]. The study was performed in accordance with the Declaration of Helsinki.

Cells and reagents

DLBCL cell lines (n = 30) were purchased from ATCC and kind gifts of MD Anderson Cancer Center laboratories (Drs. Pham and Ford, ref. [7,8]). Targeted next-generation sequencing, biomarker expression and FISH analysis were performed in these lines. Selinexor (KPT-330) was purchased from Selleckchem (Houston, Texas, USA). INCB057643 was kindly provided by Incyte Corporation.

Tissue microarray (TMA) immunohistochemistry (IHC) analysis

Biopsies obtained prior to immuno-chemotherapy were processed according to standardize procedures as described elsewhere [3-6] and TMA was constructed using tissue array. IHC analysis was conducted on TMA sections with the following antibodies: XPO1 (Abcom, clone ab24189), MYC (clone Y69, Epitomics, Burlingame, CA), BCL2 (clone 124, DAKO, Carpinteria, CA), and p53 (DO-7, DAKO, Carpinteria, CA). Expression level of these proteins for each case were determined through calculating the percentage of positive cells in TMA cores, combining with their staining intensity. The high expression cutoffs for biomarkers were determined by the mean expression levels and prognostic analysis in the study cohort

according to the X-tile statistical software (http://www.tissuearray.org/rimmlab): >30% for XPO1, ≥70% for MYC [9], ≥70% for BCL2 [5], and >10% for p53 [6].

Cell viability assay

DLBCL/HGBCL cell lines were separately plated at 5000-10000 cells per well in 384-well plates. Treatment with single or combination drugs for 72 hours, cell viabilities were assessed by the CellTiter-Glo Luminescent Cell Viability Assay, following the manufacturer's instructions (Promega, Madison, WI).

Apoptosis assay

Annexin V/PI (Ebioscience, San Diego, USA) dual-staining and Flow cytometry analysis were used to measured cell apoptosis according to the manufacturer's instructions. Briefly, after exposure with selinexor alone or combined with INCB057643 for 48 hours, cells were collected and washed by iced PBS, stained with Annexin V/PI reagent for 15 min in the dark at 4°C, and then examined with flow cytometry of a FACS canto (BD, Oxford, UK).

Reference

1. Swerdlow SH, Campo E, Pileri SA, Harris NL, Stein H, Siebert R, et al. The 2016 revision of the World Health Organization classification of lymphoid neoplasms. Blood. 2016; 127(20): 2375-2390.

2. Campo E, Swerdlow SH, Harris NL, Pileri S, Stein H, Jaffe ES. The 2008 WHO classification of lymphoid neoplasms and beyond: evolving concepts and practical applications. Blood. 2011; 117(19): 5019-5032.

3. Visco C, Li Y, Xu-Monette ZY, Miranda RN, Green TM, Li Y, et al. Comprehensive gene expression profiling and immunohistochemical studies support application of immunophenotypic algorithm for molecular subtype classification in diffuse large B-cell lymphoma: a report from the

International DLBCL Rituximab-CHOP Consortium Program Study. *Leukemia* 2012; **26**(9): 2103-2113.

4 Xu-Monette ZY, Wu L, Visco C, Tai YC, Tzankov A, Liu WM, et al. Mutational profile and prognostic significance of TP53 in diffuse large B-cell lymphoma patients treated with R-CHOP: report from an International DLBCL Rituximab-CHOP Consortium Program Study. Blood. 2012;120(19): 3986-3996.

5. Hu S, Xu-Monette ZY, Tzankov A, Green T, Wu L, Balasubramanyam A, et al. MYC/BCL2 protein coexpression contributes to the inferior survival of activated B-cell subtype of diffuse large B-cell lymphoma and demonstrates high-risk gene expression signatures: a report from The International DLBCL Rituximab-CHOP Consortium Program. Blood. 2013;121(20):4021-4031; quiz 4250.

6. Xu-Monette ZY, Moller MB, Tzankov A, Montes-Moreno S, Hu W, Manyam GC, et al. MDM2 phenotypic and genotypic profiling, respective to TP53 genetic status, in diffuse large B-cell lymphoma patients treated with rituximab-CHOP immunochemotherapy: a report from the International DLBCL Rituximab-CHOP Consortium Program. Blood. 2013; 122:2630–2640.

7. Pham LV, Fu L, Tamayo AT, Bueso-Ramos C, Drakos E, Vega F, et al. Constitutive BR3 receptor signaling in diffuse, large B-cell lymphomas stabilizes nuclear factor-kappaB-inducing kinase while activating both canonical and alternative nuclear factor-kappaB pathways. Blood. 2011; 117(1): 200-210.

8. Li L, Zhang J, Chen J, Xu-Monette ZY, Miao Y, Xiao M, et al. B-cell receptor–mediated NFATc1 activation induces IL-10-STAT3-PD-L1 signaling in diffuse large B-cell lymphoma. Blood. 2018; 132(17):1805-1817.

9. Xu-Monette ZY, Dabaja BS, Wang X, Tu M, Manyam GC, Tzankov A, et al. Clinical features, tumor biology, and prognosis associated with MYC rearrangement and Myc overexpression in diffuse large B-cell lymphoma patients treated with rituximab-CHOP. Mod Pathol. 2015; 28(12):1555-1573.

Table S1. Clinicopathologic and molecular characteristics of studied DLBCL patients (n = 544) with high or low XPO1 expression

	DLBCL		
	**XPO1^{low}**	**XPO1^{high}**	
**Characteristic**	n	n	*P*
**Age**			
≤60 years	157	77	0.64
>60 years	214	96	
**Sex**			
Male	215	100	1.0
Female	156	73	
**Stage**			
I-II	170	78	0.85
III-IV	187	90	
**B symptoms**			
No	227	107	0.84
Yes	122	60	
**Serum LDH level**			
Normal	135	58	0.38
Elevated	202	104	
**No. of extranodal sites**			
0 or 1	276	123	0.37
≥2	79	43	
**ECOG performance status**			
0 or 1	277	127	0.25
≥2	51	32	
**Largest tumor size**			
<5 cm	154	78	0.75
≥5 cm	117	54	
**IPI score**			
0-2	222	104	0.85
3-5	134	66	
**Therapy response**			
CR	289	117	0.19
Non-CR	82	56	
***MYC* rearrangement**			
No	227	102	0.31
Yes	27	17	
***BCL2* rearrangement**			
No	252	110	1.0
Yes	54	24	
***MYC*/*BCL2* double-hit**			
No	283	127	0.76
Yes	9	3	
**MYC^{high}BCL2^{high}**			
No	298	137	1.0
Yes	66	31	
***TP53* mutation**			

No	261	111	0.40
Yes	69	36	
**p53 expression**			
≤10%	218	77	**0.006**
>10%	101	64	
**MYC expression**			
<70%	249	102	0.09
≥70%	110	64	
**MYC^{high}p53^{+}**			
No	308	126	**0.005**
Yes	38	33	

Abbreviations: DLBCL, diffuse large B-cell lymphoma; LDH, lactate dehydrogenase; ECOG, Eastern Cooperative Oncology Group; IPI, International Prognostic Index; CR, complete response.

Table S2. Significantly differentially expressed genes between $XPO1^{high}$ and $XPO1^{low}$ DLBCL patients with concurrent *TP53* mutation and high MYC expression (false discovery rate: 0.20)

**$XPO1^{high}$ vs $XPO1^{low}$**		
**Function**	**Upregulated**	**Downregulated**
CD47 receptor	*SIRPA*	
Redox regulation	*C10orf58*	
DNA repair, cell cycle, checkpoint regulation		*RBBP8*
Histone, centrosome		*H2AFV*, *CSPP1*
RNA helicase, splice factor	*DDX43*	*ZRANB2*
Mitochondria DNA replication		*C10orf2*
Protein modification		*MAP1D*
Growth factor, biosynthesis		*PIGF*
Calcium signaling		*CAMK2D*
Unknown function	*TMEM145*, *LOC643988*	

Supplementary Figure legend

Figure S1. Biomarker study for XPO1 and selinexor. **(A-B)** $XPO1^{high}$ expression showed significant adverse prognostic impact in the ABC subtype but not the GCB subtype of DLBCL. **(C)** $XPO1^{high}$ expression showed a trend of unfavorable prognostic effect on PFS in *MYC*-rearranged (*MYC*-R^{+}) DLBCL. **(D)** $XPO1^{high}$ expression was associated with significantly poorer survival in DLBCL patients with wild-type (Wt) *TP53*. **(E)** ABC-DLBCL and GCB-DLBCL cells showed similar sensitivity to the cytotoxicity of selinexor. **(F)** *TP53* mutation (Mut-*TP53*) significantly reduced the anti-lymphoma efficacy of selinexor in HGBCL-DH cells. IC50 values were calculated by GraphPad Prism 8 based on the cell viability data after 72-hour treatment.

Figure S1

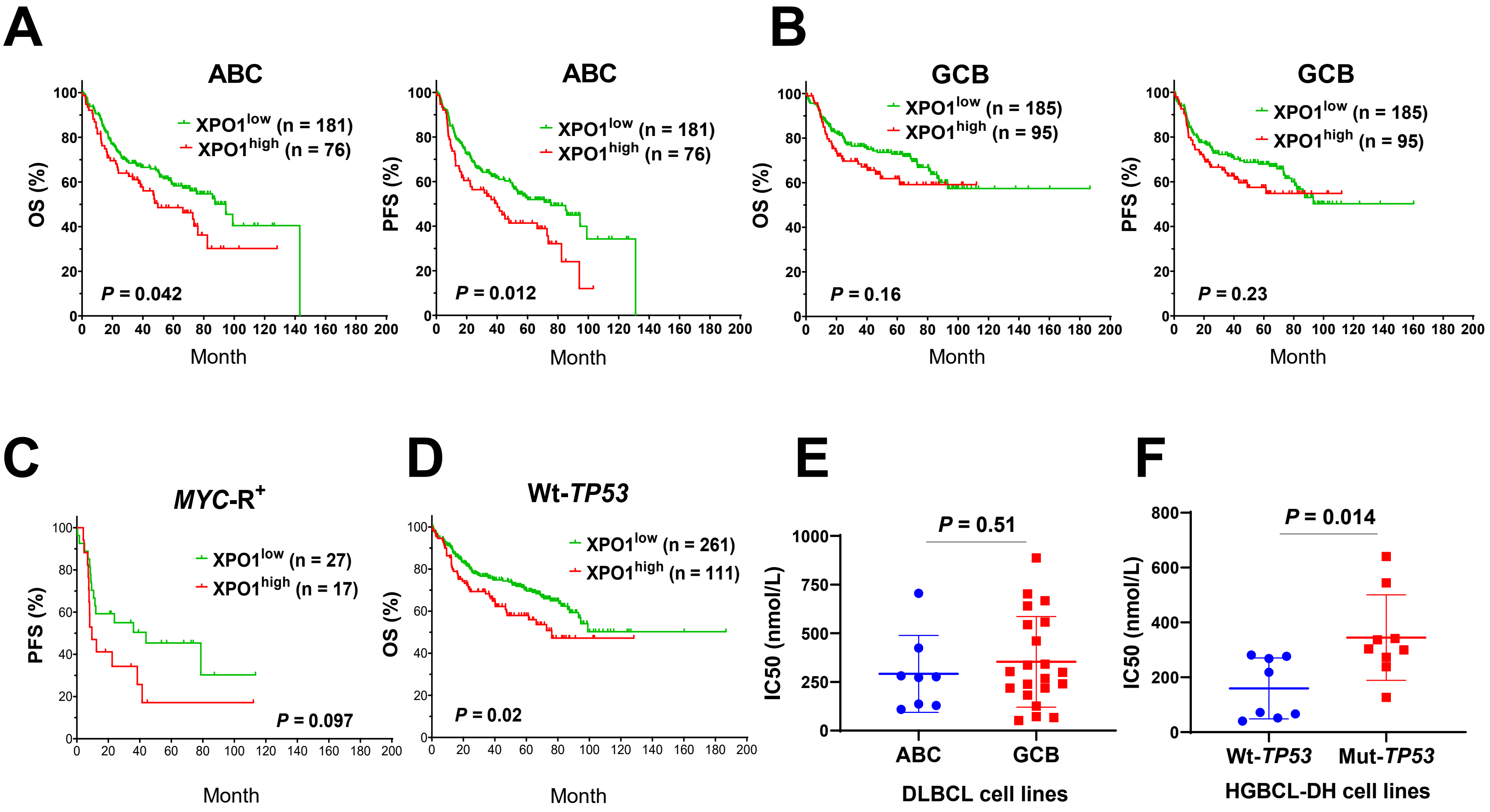
A
ABC
XPO1low (n = 181)
XPO1high (n = 76)
OS (%)
P = 0.042
Month
ABC
XPO1low (n = 181)
XPO1high (n = 76)
PFS (%)
P = 0.012
Month
B
GCB
XPO1low (n = 185)
XPO1high (n = 95)
OS (%)
P = 0.16
Month
GCB
XPO1low (n = 185)
XPO1high (n = 95)
PFS (%)
P = 0.23
Month
C
MYC-R+
XPO1low (n = 27)
XPO1high (n = 17)
PFS (%)
P = 0.097
Month
D
Wt-TP53
XPO1low (n = 261)
XPO1high (n = 111)
OS (%)
P = 0.02
Month
E
P = 0.51
IC50 (nmol/L)
ABC
GCB
DLBCL cell lines
F
P = 0.014
IC50 (nmol/L)
Wt-TP53
Mut-TP53
HGBCL-DH cell lines
